# *QuickStats*: Age-Adjusted Suicide Rates* for the Three Leading Methods of Suicide, by Race and Ethnicity^†^ — National Vital Statistics System, United States, 2020

**DOI:** 10.15585/mmwr.mm7130a6

**Published:** 2022-07-29

**Figure:**
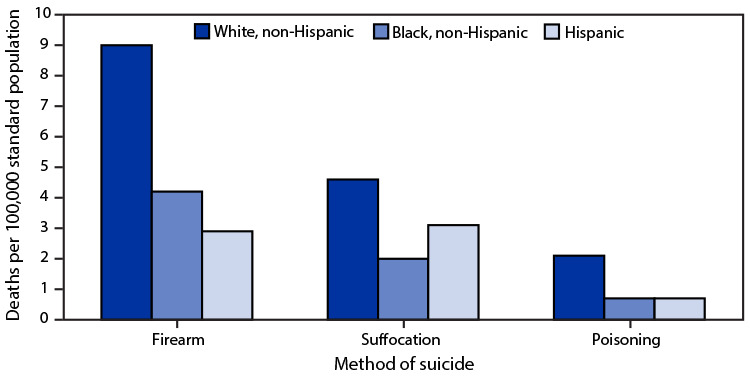
Age-adjusted rates for all three leading methods of suicide (firearm, suffocation, and poisoning) were highest for non-Hispanic White (White) persons compared with non-Hispanic Black (Black) and Hispanic or Latino (Hispanic) persons. The age-adjusted rate of suicide by firearm was 9.0 per 100,000 standard population for White persons followed by 4.2 for Black persons and 2.9 for Hispanic persons. The rate of suicide by suffocation (includes hanging) was 4.6 for White persons followed by 3.1 for Hispanic persons and 2.0 for Black persons. The rate of suicide by poisoning was 2.1 for White persons and 0.7 for both Black and Hispanic persons. Suicide by firearm was the leading method for both White and Black persons, whereas suffocation was the leading method for Hispanic persons followed closely by firearm.

**Source**: National Vital Statistics System, Mortality Data. http://www.cdc.gov/nchs/nvss/deaths.htm

For more information on this topic, CDC recommends the following link: https://www.cdc.gov/suicide

